# Transition state analogue of MTAP extends lifespan of APC^Min/+^ mice

**DOI:** 10.1038/s41598-021-87734-6

**Published:** 2021-04-23

**Authors:** Ross S. Firestone, Mu Feng, Indranil Basu, Karina Peregrina, Leonard H. Augenlicht, Vern L. Schramm

**Affiliations:** 1 Department of Biochemistry, Albert Einstein College of Medicine, Bronx, NY 10461 USA; 2 Department of Radiation Oncology, Albert Einstein College of Medicine, Bronx, NY 10461 USA; 3 Department of Cell Biology, Albert Einstein College of Medicine, Bronx, NY 10461 USA; 4 Present Address: Department of Medicine, Icahn School of Medicine at Mount Sinai, New York, NY 10029 USA

**Keywords:** Enzyme mechanisms, Enzymes, Metabolomics, Oncology, Cancer

## Abstract

A mouse model of human Familial Adenomatous Polyposis responds favorably to pharmacological inhibition of 5′-methylthioadenosine phosphorylase (MTAP). Methylthio-DADMe-Immucillin-A (MTDIA) is an orally available, transition state analogue inhibitor of MTAP. 5′-Methylthioadenosine (MTA), the substrate for MTAP, is formed in polyamine synthesis and is recycled by MTAP to *S*-adenosyl-l-methionine (SAM) via salvage pathways. MTDIA treatment causes accumulation of MTA, which inhibits growth of human head and neck (FaDu) and lung (H359, A549) cancers in immunocompromised mouse models. We investigated the efficacy of oral MTDIA as an anti-cancer therapeutic for intestinal adenomas in immunocompetent APC^Min/+^ mice, a murine model of human Familial Adenomatous Polyposis. Tumors in APC^Min/+^ mice were decreased in size by MTDIA treatment, resulting in markedly improved anemia and doubling of mouse lifespan. Metabolomic analysis of treated mice showed no changes in polyamine, methionine, SAM or ATP levels when compared with control mice but indicated an increase in MTA, the MTAP substrate. Generation of an MTDIA-resistant cell line in culture showed a four-fold amplification of the methionine adenosyl transferase (*MAT2A*) locus and expression of this enzyme. MAT2A is downstream of MTAP action and catalyzes synthesis of the SAM necessary for methylation reactions. Immunohistochemical analysis of treated mouse intestinal tissue demonstrated a decrease in symmetric dimethylarginine, a PRMT5-catalyzed modification. The anti-cancer effects of MTDIA indicate that increased cellular MTA inhibits PRMT5-mediated methylations resulting in attenuated tumor growth. Oral dosing of MTDIA as monotherapy has potential for delaying the onset and progression of colorectal cancers in Familial Adenomatous Polyposis (FAP) as well as residual duodenal tumors in FAP patients following colectomy. MTDIA causes a physiologic inactivation of MTAP and may also have efficacy in combination with inhibitors of MAT2A or PRMT5, known synthetic-lethal interactions in *MTAP*^−/−^ cancer cell lines.

## Introduction

Colorectal cancer (CRC) is the third leading cause of cancer-related death in the United States^1^, despite a 50% decreased incidence from colonoscopy and polypectomy^2^. Approximately 80% of malignant CRCs in humans exhibit mutations in *APC*, *KRAS* and *p53*, part of the adenoma-carcinoma sequence that drives normal colonic epithelium to progress to adenomas and eventually to malignant carcinomas^3^. Familial Adenomatous Polyposis (FAP) is an autosomal dominant disorder in which affected individuals inherit a heterozygous mutation in *APC*, resulting in an increased predisposition to developing CRC, even at relatively young ages^4,5^.

The APC^Min/+^ mouse model is a well-characterized model of human FAP. It involves a loss of function mutation in the *APC* locus, leading to a genetic predisposition to intestinal adenoma formation in mice^6^. The Min (Multiple Intestinal Neoplasia) mutation in *APC* is a nonsense mutation at codon 850 of the *APC* gene that results in a significant truncation of the expressed protein^7^. APC is critical for facilitating the phosphorylation, and subsequent degradation of β-catenin in the Wnt signaling pathway. The Min mutation results in a loss of APC function, causing activation of the Wnt signaling pathway, thereby initiating polyp formation in intestinal epithelium^8^. APC^Min/+^ mice differ from humans with FAP, as polyps form primarily in the small intestine of APC^Min/+^ mice, while in humans, polyps form primarily in the colon. Nevertheless, the APC^Min/+^ model is a valuable tool to investigate the efficacy of novel treatments for spontaneously forming CRC, for FAP, and for the tumors that develop in the small intestine of FAP patients following colectomy to eliminate extensive large intestinal tumors^9^.

**Figure 1:**
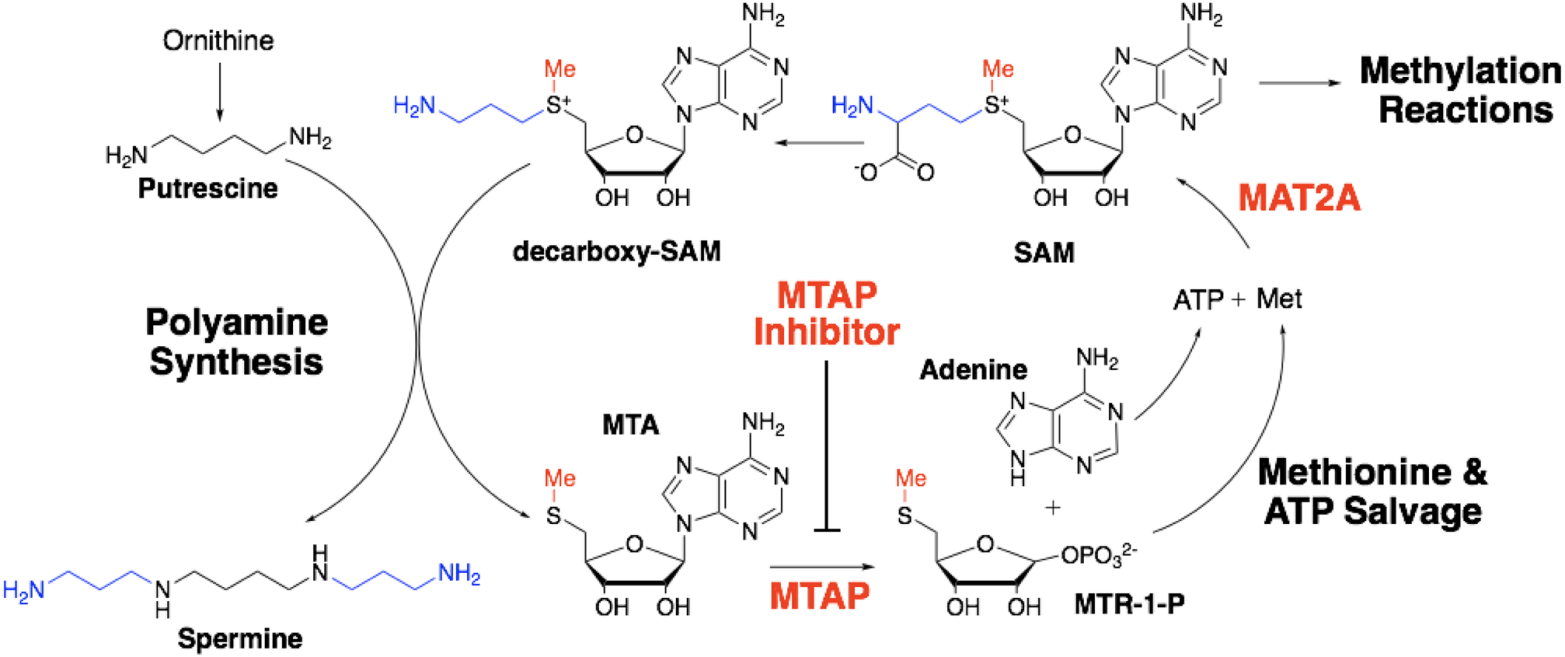
The role of 5′-methylthioadenosine phosphorylase (MTAP) in the context of methionine metabolism and polyamine synthesis. MTAP catalyzes the phosphorolysis of 5′-methylthioadenosine (MTA) to form adenine and 5-methylthio-α-d-ribose 1-phosphate (MTR-1-P), which can be recycled to form ATP and methionine followed by conversion to S-adenosyl-l-methionine (SAM) by the MAT2A-catalyzed reaction. The S-methyl of SAM (red) is conserved in the MTA salvage reaction enabled by MTAP action. MTAP inhibitors interfere with MTA metabolism leading to elevated MTA concentration in cells and growth disruption.

5′-Methylthioadenosine phosphorylase (MTAP) catalyzes the phosphorolysis of 5′-methylthioadenosine (MTA), a product of *S*-adenosyl-l-methionine (SAM)-mediated polyamine synthesis, into adenine and 5-methylthio-α-d-ribose 1-phosphate (MTR). MTAP has been identified as an anti-cancer target^10^. Recycling of adenine into ATP and MTR into methionine permits resynthesis of SAM via methionine adenosyl transferases (i.e. MAT2A) (Fig. 1). The transition-state structure of MTAP has been solved and used to develop transition-state analogue inhibitors of the enzyme. One such inhibitor, Methythio-DADMe-Immucillin-A (MTDIA), is an 86 pM transition-state analogue inhibitor of MTAP. It has demonstrated anti-cancer properties in selected in vitro cancer cell lines and in several in vivo mouse models of human cancers. Mouse xenografts of FaDu head and neck cancer and A549 and H358 lung cancers in immunocompromised mice are susceptible to oral treatment with MTDIA^11,12^. However, there is no information regarding the efficacy of MTDIA in cancer therapy in immunocompetent mice with spontaneously forming cancers. Moreover, although the MTDIA target has been identified, the mechanism of cancer cell growth inhibition remains under investigation.

Despite being an anti-cancer target, *MTAP* is genomically deleted in 15% of human cancers together with the neighboring *CDKN2A*, one of the most frequently deleted tumor suppressor genes in human cancers. Gastrointestinal cancers have varying levels of MTAP homozygous deletions, including 17% prevalence in gastrointestinal stromal tumors, 12% in gastric carcinoma, 22% in esophageal carcinoma, and as low as 1–2% in CRC^13,14^. Although MTAP deletion frequency is more rare in CRC than in other cancers, some CRC cell lines have been shown to have significantly increased MTAP and MAT2A expression, indicating increased reliance on these metabolic pathways^15–17^. MTAP^−/−^ cancer cell lines have increased levels of MTA, which inhibit the activity of the methyltransferase PRMT5, as a 260 nM inhibitor^18^. Partial inhibition of PRMT5 by elevated MTA levels enhances vulnerability to inhibition of other targets involved in histone, DNA and intron methylation, including PRMT5 itself as well as MAT2A, the enzyme responsible for SAM synthesis, essential for methylation reactions^19,20^. More recently, type 1 PRMT and PRMT5 inhibitors were shown to synergize in MTAP^−/−^ cancers^21^. Thus, inhibition of MTAP in cancers by a specific inhibitor has potential as a sole agent and as an enhancer of new agents designed to target histone, DNA, and intron methylation.

Here, the efficacy of MTDIA as an anti-tumor therapeutic is investigated in the APC^Min/+^ mouse model of CRC. The physiologic impact of MTDIA on tissue metabolite levels and arginine methylation were also determined, since elevated MTA can potentially interact with other targets including type 1 PRMTs, PRMT5 and MAT2A by mimicking the metabolic physiology observed in MTAP^−/−^ cancer cell lines. Finally, the mechanism of action of MTDIA was investigated by assessing PRMT5 activity in treated mice and by generation of an MTAP inhibitor-resistant cell line of FaDu cancer cells, revealing amplification of MAT2A, a known target in MTAP^−/−^ cancers.

## Material and methods

### Animal husbandry

All methods were carried out in accordance with relevant guidelines and regulations. All animal studies were carried out in compliance with ARRIVE guidelines.

Metabolic studies and genetically driven mouse models of CRC used *C57BL/6J* wild-type (WT) and *C57BL/6J-ApcMin/J* (APC^Min/+^) mice purchased from Jackson Laboratory and housed in the Barrier Facility of the Albert Einstein College of Medicine. All experimental procedures were conducted under protocols approved by the Einstein IACUC. Mice were fed 5058 diet from LabDiet (Chow) during breeding and strain maintenance. During experiments, mice were fed purified AIN76A diet from Research Diets Inc. Experimental mice were generated by crossing APC^Min/+^ males and WT females. Offspring were genotyped via PCR, and then weaned at 21 days after birth to the AIN76A diet.

For PCR genotyping, DNA was isolated from tail clips using DNeasy Blood and Tissue Kits (Qiagen). PCR analysis for Apc genotype used a forward primer for the wild-type allele (GCCATCCCTTCACGTTAG), a forward primer for the mutant APC^Min/+^ allele (TTCTGAGAAAGACAGAAGTTA) and a common reverse primer (TTCCACTTTGGCATAAGGC).

### Drug preparation

Methylthio-DADMe-Immucillin-A (MTDIA) was synthesized as previously described and provided by the Ferrier Research Institute (Wellington, NZ)^22,23^. MTDIA was dissolved in sterile drinking water and its concentration determined by spectrophotometry (MTDIA ε_275_ = 8.5 cm^-1^ mM^−1^). The average dose was calibrated to the water consumption of C57BL6/J mice at each respective dose.

### Survival study

Following weaning at 21 days, APC^Min/+^ mice (N = 41) were divided into four treatment groups for Study A (Figure S1). The control group received sterile water (N = 11), the 10 mg/kg/day (N = 10), 20 mg/kg/day (N = 10), and 30 mg/kg/day (N = 10) dosage groups received sterile water with MTDIA (phosphate salt) dissolved to achieve concentrations for appropriate dosing in their drinking water. Mouse sex was distributed roughly equally among treatment groups. Mice were fed AIN76A diet from Research Diets Inc., throughout the experiments^24,25^. Mice were monitored throughout the experiment and days to death recorded^26^. Statistical significance of survival data was assessed using the Gehan-Breslow-Wilcoxon test^27,28^.

### Histopathology, metabolomics, and blood analysis

After weaning at 21 days, APC^Min/+^ mice were randomized to a control group that received no MTDIA, and a treatment group receiving 20 mg/kg/day MTDIA administered orally (Study B, Figure S1). Mice were monitored until 150 days old (129 treatment days), predicted to be shortly prior to the death of control mice determined from survival studies.

Mice were euthanized by CO_2_ asphyxiation followed by cervical dislocation. Blood was collected by cardiac puncture for analysis of a panel of metabolic parameters including basic metabolic panels (BMP), liver function tests (LFT) and complete blood counts (CBC). Statistical analysis of blood studies was performed using a two-tailed Student’s t-test. Gastrointestinal (GI) tissue was removed, flushed with phosphate buffered saline and prepared as “Swiss-rolls” for histopathologic analysis^29,30^. Mouse livers were dissected for metabolomic analysis.

Formalin fixed, paraffin embedded (FFPE) Swiss rolled tissue encompassing the entire intestine from the duodenum through the colon were stained with hematoxylin and eosin (H&E). Number, size, and histopathology of tumors were assessed by light microscopy.

Immunohistochemical (IHC) analysis for SDMA on FFPE tissue employed an anti-SDMA primary antibody (1:400, Cat. # MBS619480, MyBioresource)^31^ followed by a biotin-conjugated secondary antibody (1:100, BA-1000, Vector Lab.). For visualization we used an Avidin–Biotin enzymatic complex based detection followed by hematoxylin counterstaining. Signal intensity within intestinal epithelial cells was determined by measuring the percentage of positively stained areas in several regions of interest (ROIs) selected for each mouse (details in SI section C4). The mean signal intensity among ROIs for each individual mouse was compared, and statistical analysis was performed using a two-tailed Student’s t-test.

Liver tissue was frozen in liquid nitrogen and analyzed by Human Metabolome Technologies, Inc., to measure the concentrations of 117 metabolites, including common metabolic carbohydrates, amino acids, and nucleic acid precursors (details in SI section C2). An additional cohort of APC^Min/+^ mice, receiving 30 mg/kg/day oral MTDIA was included in this study to assess dose-dependent effects on metabolomic parameters.

### Assessing MTDIA and MTA toxicity

Six to eight-week old CD1-stock female mice (Charles River Laboratories (MA)) were used to assess MTDIA and MTA toxicity. A total of 40 animals (5 in each of 8 groups) were evaluated. Mice were fed laboratory rodent diet 5001 (LabDiet, MA) and autoclaved drinking water, and in experimental groups were dosed daily via intraperitoneal (IP) injections for 28 days using 154.5 mM MTDIA and/or 13 mM MTA solutions according to Table S3. Mice were monitored daily and body weights were recorded weekly. After the 28th day, blood samples were collected via cardiac puncture using isoflurane anesthesia and submitted to Antech GLP for CBC and BMP analysis. Necropsies were conducted on all found-dead, moribund- and schedule-sacrificed animals. The necropsies included gross examination of the external surface of the body, all orifices, the cranial, thoracic, abdominal, and pelvic cavities and their contents. Tissues and organs from scheduled- and moribund-sacrificed animals were processed for histopathology. Weights of fixed tissue were taken of the heart, liver, kidneys, and brain at the time of necropsy. Histopathologic evaluations were conducted on all protocol-designated organs and tissues from all animals in the control and high dose groups. Tissues were fixed in formalin and were embedded in paraffin for histopathological analysis using H&E staining.

### Generation of MTDIA-resistant FaDu cells and genomic assessment

FaDu cells were provided by Dr. M. B. Prystowsky as previously described^11^. FaDu cell culture was selected to generate an MTDIA-resistant cell line, as previous reports demonstrated it to be sensitive to the inhibitor in the presence of MTA. Growth of FaDu cells in culture is not inhibited by 1 µM MTDIA alone or by 20 µM MTA alone, but the combination of MTDIA with MTA (20 µM) induces apoptotic cell death with an MTDIA IC_50_ of 50 nM^11,32^. An MTDIA-resistant FaDu cell line was selected by culturing cells in Eagle’s media supplemented with 10% fetal bovine serum, 100 units/mL penicillin, 100 mg/mL streptomycin, 0.1 mM nonessential amino acids, 1 mM sodium pyruvate and increasing concentrations of MTA (from 5 to 20 µM) and MTDIA (from 0.5 to 1 µM). Cells were passaged and media was changed every 3 days over a period of 102 days (Details in SI section C3). After resistant FaDu (FaDu-R) cells were obtained, they were cultured for eight passages in culture medium containing 1 µM MTDIA and 20 µM MTA.

Genomic DNA was isolated from FaDu parental and FaDu-R cells by DNAeasy (Qiagen) and analyzed by massively parallel sequencing based on the Illimina Hi Seq 2000 platform with paired end-read and 100 bp read lengths. Genomic analysis was performed by comparing DNA sequences with a 200 bp genomic library constructed from the parental DNA. The average number of reads from the DNA of each cell type was sixteen. The relative numbers of reads from each probe complimentary to genomic DNA were compared for native and resistant cell lines. The ratio of reads from parental FaDu and FaDu-R were determined as a function of DNA region (Figure S4).

MAT2A and its associated regulatory protein MAT2B expression were quantified via Western blotting of extracts from parental FaDu cells and FaDu-R cells using β-actin as a standard to assess if copy number changes resulted in differential protein expression. Additionally, quantitative polymerase chain reaction analyses were used to measure the cellular content of mRNA for *MAT2A* using β-actin and β_2_-microglobulin as mRNA standards.

## Results

### Survival study

**Figure 2:**
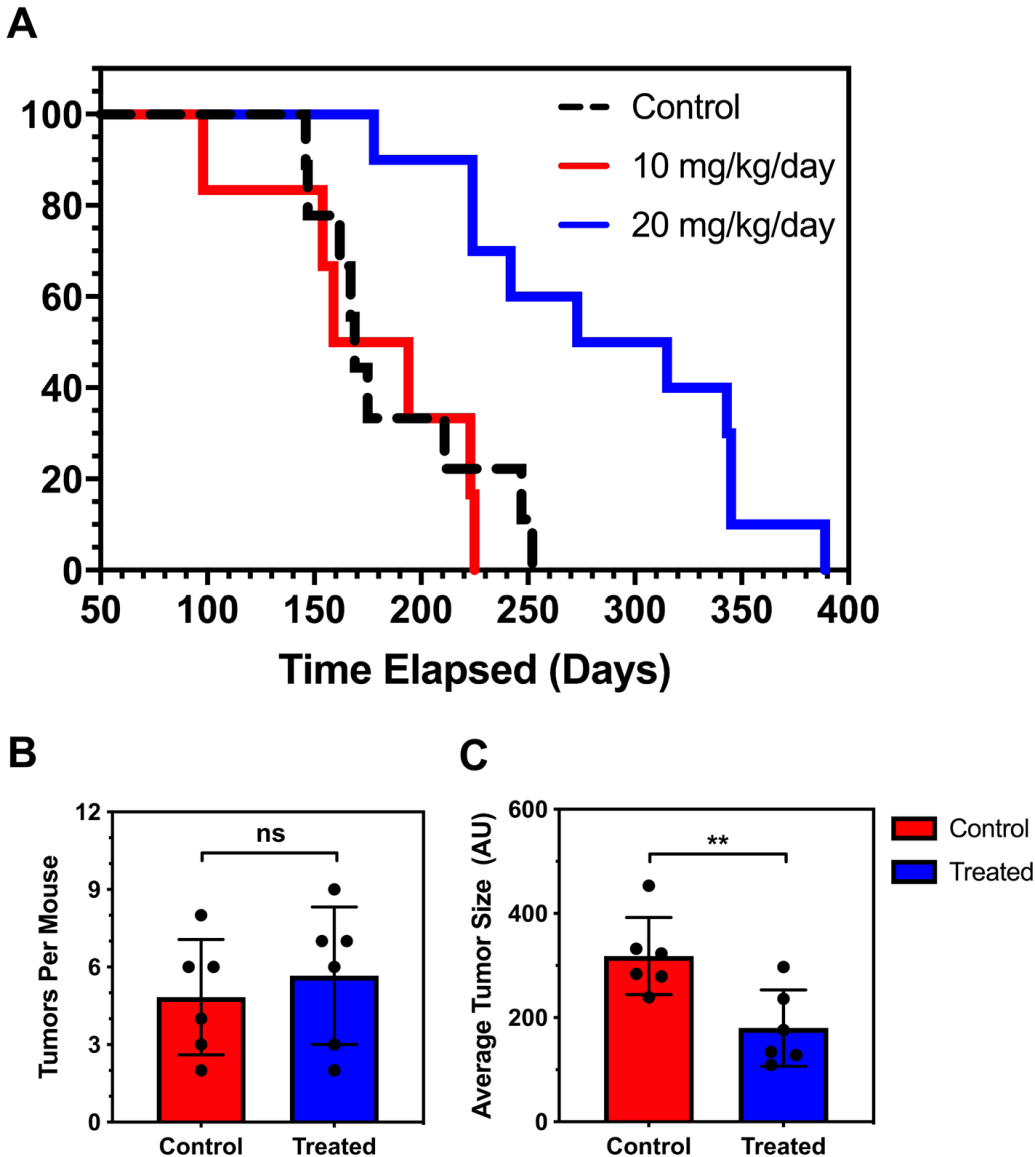
(**A**) Kaplan–Meier analysis of APC^Min/+^ mouse survival (Study A). The majority of APC^Min/+^ untreated mice (N = 11) die before 170 days of life, consistent with prior reports^30^. Oral MTDIA dosing at 10 mg/kg/day (N = 10) showed no significant effect on mouse survival. Oral MTDIA dosing at 20 mg/kg/day (N = 10) showed an approximate doubling in lifespan for APC^Min/+^ mice. (**B,C**) Histolopathologic assessment of APC^Min/+^ mouse intestinal tissue. The mean number of tumors (**B**) and average size (**C**) of intestinal adenomas was compared in control (N = 6) and 20 mg/kg/day treated (N = 6) mice. The change in the number of tumors per mouse following treatment was not statistically significant. The average tumor size for treatment mice was decreased by 43% in mice treated with 20 mg/kg/day MTDIA (*p* = 0.0086). Tumor size was measured in “AU” or arbitrary units.

Apc^Min/+^ mice develop multiple intestinal tumors that cause bleeding and intestinal blockage and kill the mice within several months of birth, but tumor phenotype can vary in different colonies. Here, the median survival of untreated control Apc^Min/+^ mice was 169 days (Fig. 2A, Table S1), consistent with previous reports^33^. Mice treated with 10 mg/kg/day MTDIA showed no statistically significant improvement in survival with median survival of 176.5 days (*p* = 0.7). However, mice treated with 20 mg/kg/day showed a highly significant improvement in lifespan with median survival of 294 days (*p* = 0.001 relative to control mice). Mice treated with 30 mg/kg/day showed a median survival of 226 days (*p* = 0.04 relative to control mice). There were no significant differences in survival when stratifying mice by sex. Thus, 20 mg/kg/day provides an effective dose for prolonged survival in APC^Min/+^ mice. To further investigate this, the histopathology of the intestine was investigated. MTDIA did not alter tumor number, but tumor size was significantly decreased by 43% (*p* = 0.0086), (Fig. 2B,C). There were no significant differences when stratifying this tumor histologic data by mouse sex. Thus, MTDIA appears to inhibit tumor growth rather than tumor initiation.

### Metabolomic effects of MTDIA therapy

**Figure 3:**
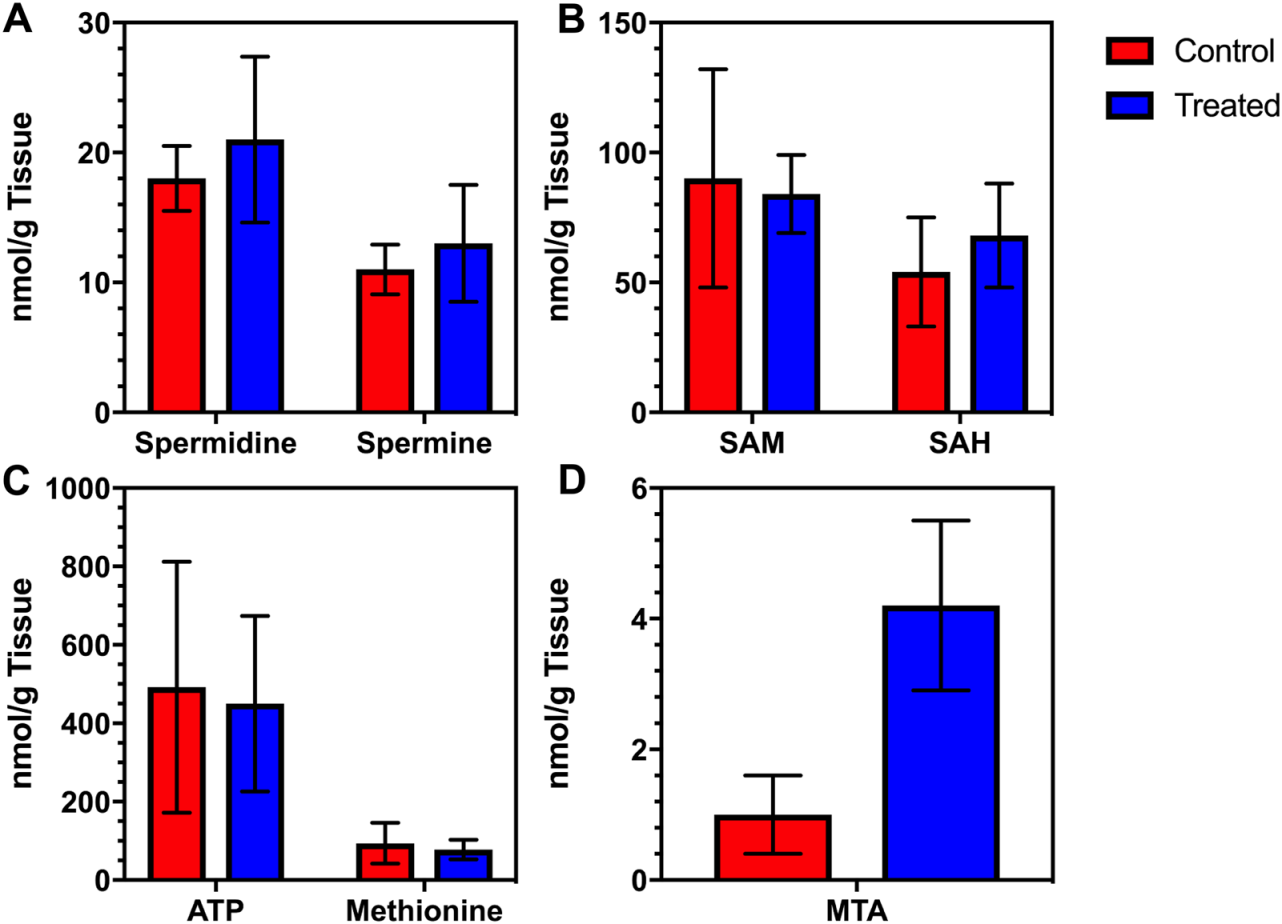
Selected liver metabolites from APC^Min/+^ mice. Untreated (N = 6) and treated mice (N = 6) receiving 20 mg/kg/day MTDIA following weaning were euthanized at day 150 and livers were extracted to analyze metabolites. No significant changes were noted in polyamine levels (**A**), SAM or SAH levels (**B**), or ATP and methionine levels (**C**). A 4.2-fold increase in MTA was observed in mice receiving 20 mg/kg/day oral MTDIA (*p* = 0.001) (**D**). The anti-cancer effect of MTAP inhibition is coincident with MTA accumulation. Expanded metabolomics data is shown in the SI, Table S12.

Metabolic changes induced by oral administration of MTDIA (Study B) indicated no significant changes in polyamines (spermidine, spermine, putrescine), ATP, methionine, SAM or *S*-adenosyl-l-homocysteine (SAH) in comparison of treated and untreated mice (Fig. 3A–C, Table S12). Thus, pathways of polyamine synthesis, ATP, methionine salvage, and total methylation potential were not affected by MTAP inhibition (Fig. 1). In contrast, cellular levels of MTA were increased four-fold as a result of MTDIA treatment (Fig. 3D), from 1.0 ± 0.6 nmol/g liver tissue in untreated APC^Min/+^ mice to 4.2 ± 1.3 nmol/g liver tissue in APC^Min/+^ mice treated with 20 mg/kg/day MTDIA (*p* = 0.001).

MTA levels in treated APC^Min/+^ mice were the same at doses of MTDIA at 20 and 30 mg/kg/day, suggesting target saturation at the lower dose. Mice treated with 30 mg/kg/day MTDIA had MTA levels of 4.2 ± 0.7 nmol/g (*p* = 0.94 relative to the 20 mg/kg/day group).

The four-fold increase in cellular MTA is similar to the increase in MTA observed in MTAP^−/−^ cancer cell lines when compared to MTAP^+/+^ isogenic lines^16,17^, indicating that in this mouse model, MTDIA therapy results in a physiologic mimic of an MTAP genetic deletion.

### Effects of MTDIA therapy on blood parameters

APC^Min/+^ mice treated with MTDIA showed no significant changes in the basic metabolic panel (BMP) or liver function parameters (LFT, Table 1). However, when comparing untreated APC^Min/+^ mice to mice treated with 20 mg/kg/day MTDIA, both groups showed hypernatremia, hyperkalemia, hyperchloremia, uremia, hypoalbuminemia, hypoproteinemia and elevated liver enzymes (aspartate aminotransferase only) when compared to normal C57BL/6 mice^34^. These values are indicative of the illness of APC^Min/+^ mice, as indicated by the shortened lifespan of both the control and treated groups of mice, compared to the 2–3 year lifespan of wild-type mice^35^.

**Table 1:** Blood, metabolite and liver function results from APC^Min/+^ mice included in Study B.

	APC^Min/+^ untreated	Treated (20 mg/kg/day)	*P* value
**Complete blood count**			
WBC (10^3^/µL)	3.8 ± 2.4	5 ± 2.7	NS
Hgb (10^3^/µL)	2.6 ± 1.8	9.7 ± 1.8	**0.007**
**Basic metabolic panel**			
Sodium (mEq/L)	160 ± 20	152 ± 8	NS
Potassium (mEq/L)	9.7 ± 1.1	8.8 ± 2.2	NS
Chloride (mEq/L)	126 ± 12	120 ± 5	NS
Blood Urea Nitrogen (mg/dL)	40 ± 14	41 ± 12	NS
Creatinine (mg/dL)	0.3 ± 0.1	0.3 ± 0.1	NS
Glucose (mg/dL)	335 ± 116	428 ± 115	NS
**Liver function test**			
Calcium (mg/dL)	9.6 ± 0.6	10.1 ± 0.6	NS
Total Protein (g/dL)	3.6 ± 0.9	4.2 ± 0.9	NS
Albumin (g/dL)	1.9 ± 0.5	2.3 ± 0.5	NS
AST (IU/L)	237 ± 143	162 ± 110	NS
ALT (IU/L)	27 ± 12	12 ± 3	NS
Alkaline Phosphatase (IU/L)	27 ± 14	33 ± 15	NS

There were no statistically significant differences between the blood and liver function values between these groups. However, both groups exhibited hypernatremia, hyperkalemia, hyperchloremia, uremia, hypoalbuminemia, hypoproteinemia and AST (aspartate aminotransaminase) elevations. The blood study shows a significant reduction in hemoglobin levels of untreated APC^Min/+^ mice relative to treated mice, indicating anemia characteristic of bleeding in untreated APC^Min/+^ mice.

Importantly, untreated APC^Min/+^ mice had an average hemoglobin level of 2.6 ± 1.8 while treated mice had an average level of 9.7 ± 1.8 (*p* = 0.007), thus the profound anemia which was one of the earliest identified defining characteristic of APC^Min/+^ mice became mild in MTDIA treated mice^6^. This difference is consistent with data from the survival study and the reduced size of the tumors, which would lead to less bleeding.

### Immunohistochemical analysis of PRMT5 activity

**Figure 4:**
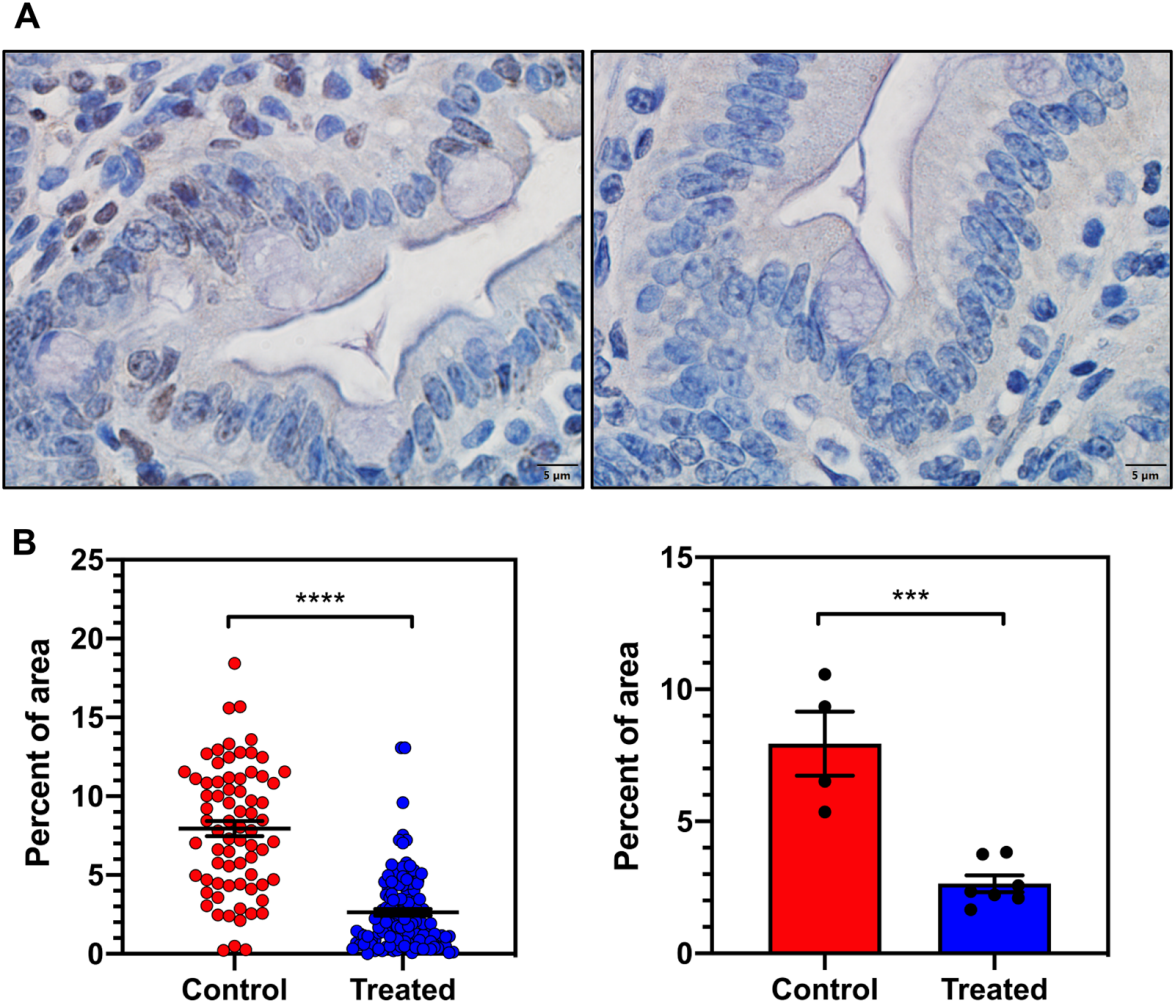
(**A**) Microscopy images (100 x) with IHC staining for symmetric dimethylarginine (SDMA) in intestinal tissue from untreated APC^Min/+^ mice (left) and those treated with 20 mg/kg/day MTDIA (right) euthanized at 150 days. SDMA signal (brown) was reduced in MTDIA-treated mice. (**B**) Quantitative data from four control and seven MTDIA-treated mice from IHC staining experiments. The left panel shows quantitative signal intensity from all ROIs among all control and treated mice with an observed statistically significant decrease in SDMA signal among MTDIA treated mice (*p* < 0.0001). The right panel shows mean ROI signal intensity for each individual mouse, with mean signal intensity three-fold less in MTDIA treated mice compared to controls (*p* < 0.001).

APC^Min/+^ mice treated with MTDIA had a significant decrease in signal on IHC analysis of intestinal tissue stained with anti-SDMA antibodies (the PRMT5 catalyzed arginine modification^36,37^). A majority of epithelial cells in the control groups displayed SDMA puncta while the staining intensity was lower in the treated groups (Fig. 4A). When comparing both individual regions of interest and the average signal intensity among several ROIs for individual mice, MTDIA treated mice showed an approximately three-fold reduction in SDMA staining relative to untreated control mice (*p* < 0.0001 for individual ROIs and *p* = 0.0004 for individual mice; Fig. 4A,B).

### Assessment of MTDIA toxicity

MTDIA toxicology studies performed on CD1 females indicated no substantive adverse effects in mice treated with intraperitoneal doses up to 317 mg/kg/day, more than 10 × the highest experimental oral dose, and more than 15 × the optimal oral therapeutic dose (Table S3). No treatment-related gross or histological lesions in vehicle- or MTDIA-treated mice were observed. Kidney, liver, spleen, thymus, heart, submandibular salivary gland, submandibular lymph node, mesenteric lymph node, lungs, trachea, esophagus, brain, ovaries, sciatic nerve, spine, spinal cord, diaphragm, aorta, adrenal gland, and cross sections of head were evaluated in all mice reaching study termination. Mice included in the experiment lost no more than 8% of their body weight over the course of the study compared to pre-treatment weights (Table S4). These well-tolerated high doses of MTDIA suggest a broad safety margin relative to the effective oral dose of 20 mg/kg/day.

### Development of an MTDIA drug-resistant cell line

Genetic resistance to drug pressure can provide insights into the mechanism of action of anti-cancer drugs. FaDu cells were selected to generate an MTDIA resistant line as they had previously been shown to be susceptible to MTDIA. In vitro, the combination of MTDIA and MTA induced apoptosis^11^. MTDIA-resistant FaDu cells (FaDu-R) were generated over 4 months of increasing drug and MTA treatment. FaDu-R cells had growth rates and microscopic morphology equivalent to the parental FaDu cell line when grown without MTDIA or MTA. Parental FaDu cells had an IC_50_ of 50 nM for MTDIA when cultured in the presence of 20 µM MTA. The FaDu-R cells grow at uninhibited rates in the presence of 1 µM MTDIA and 20 µM MTA, and are therefore at least 20-fold more resistant to MTDIA therapy than the parental strain.

Genome comparison of FaDu to FaDu-R revealed a four-fold gene amplification on chromosome 2, from 83.4 to 85.8 mb. No other regions of the genome showed a defined amplification or deletion (Figure S4). Most of the DNA in the 2.4 mb region is non-encoding. Fifteen open reading frames were identified in the amplified region, however, only *MAT2A* codes for a protein related to the pathway with MTAP involvement. The *MAT2A* gene resides in cytogenetic band 2p11.2 and includes 6,303 bases in this region with its start at 85,766,101 and its end at 85,772,403 from the p-arm terminus. The other 14 amplified genes and their functions are unrelated to MTA metabolism (Table S2).

**Figure 5:**
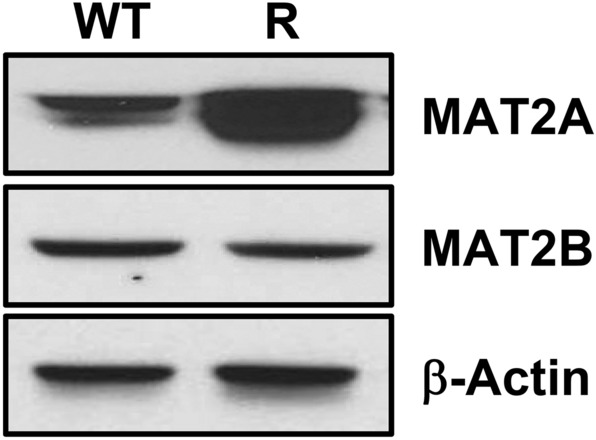
Western blot analysis comparing parental FaDu cells (WT) compared to MTDIA-resistant FaDu cells (R). MAT2A expression is increased in FaDu resistant cells while MAT2B levels are unchanged. MTDIA resistance is linked to MAT2A overexpression and increased capacity for SAM production. The doublet appearance of the MAT2A band is consistent with prior reports. Unedited data is shown in Figure S5^32^.

Quantitative PCR experiments showed a 7.3-fold increase in *MAT2A* mRNA in FaDu-R cells compared to FaDu cells when using β-actin as an mRNA standard and 4.0-fold increase when using β2-microglobulin as the mRNA standard. Western blot analysis of cellular extract shows a four-fold increase in MAT2A levels in FaDu-R cells compared to FaDu cells (Fig. 5). MAT2A appears as two isoelectric bands in the Western blots, consistent with previous reports^38^. MAT2A is an *S*-adenosylmethionine synthase, catalyzing the formation of SAM from methionine and ATP (Fig. 1). Increased expression of MAT2A in FaDu-R cells implicates MAT2A overexpression in establishing MTDIA resistance. MAT2B protein expression was unchanged in the FaDu-R cells (Fig. 5). MAT2B is reported to form a regulatory complex with MAT2A in vivo and is located at 5q34, not in the gene-amplified region of chromosome 2.

## Discussion

### MTDIA monotherapy in the APC^Min/+^ tumor model

MTDIA administered as a single oral agent demonstrated a significant anti-tumor effect in the APC^Min/+^ mouse model of human FAP. The lifespan of APC^Min/+^ mice was improved approximately two-fold as a result of MTDIA therapy (Fig. 2, Table S1), due to a reduction in tumor growth and progression, and a concomitant mitigation of the severe anemia that is a defining phenotype in APC^Min/+^ mice^6^.

The lack of MTDIA-associated toxicity in mice in doses up to 15 × the optimal therapeutic dose of 20 mg/kg/day indicates that MTDIA could be appropriate for long-term dosing. If considered for human use, MTDIA might be used as a long-term oral medication to delay CRC formation in high-risk patients such as those with FAP or related genetic syndromes predisposing to colon cancer formation. Further, FAP patients often undergo colectomy when colon tumor burden cannot be managed, but the frequent development of small intestinal tumors refractory to therapy is a major clinical problem. In the APC^Min/+^ mice, an inherited APC mutation similar to that in FAP patients causes principally small intestinal tumors, which mimics the residual human disease following colectomy for FAP in regards to both etiology and location. Thus, the efficacy of MTDIA in the APC^Min/+^ mice, coupled with its low toxicity, suggests potential efficacy in this clinical setting.

### A proposed mechanism of action for MTDIA as an anti-cancer therapy

The development and genetic analysis of a FaDu-resistant cell line (FaDu-R) provided significant insight into the mechanism of action for MTDIA, beyond its inhibition of MTAP. Original proposals for the anti-cancer effects by inhibition of MTAP proposed restricted polyamine synthesis by causing MTA to accumulate and cause product feedback inhibition on the polyamine synthases^11^. Another proposed mechanism for MTAP inhibition/deletion is the reduction of cellular SAM by disruption of MTA conversion to adenine and methylthio-α-d-ribose 1-phosphate, precursors of ATP and methionine, components for SAM salvage from MTA. However, the liver tissue levels of those metabolites are unaffected by MTDIA therapy even at high doses (Fig. 3, Table S12). Therefore, the elevated MTA concentrations observed as a consequence of MTAP inhibition with MTDIA are proposed to be a key component contributing to reduced cancer growth, a feature shown to be disruptive to colon cancer cells^15–17^. Elevated concentrations of MTA are known to inhibit PRMT5, which is essential for cancer cell growth both through its role as a histone methyltransferase and as a regulator of intron splicing^39^. When the prevalence of symmetric dimethylarginine (SDMA), a PRMT5-catalyzed arginine methylation pattern, was assessed in mouse intestinal tissue, MTDIA-treated mice had a three-fold lower presence of the modification, indicating diminished PRMT5 activity as a consequence of MTA accumulation from MTAP inhibition. A recently published study examining the effects of MAT2A inhibitors on both MTAP^−/−^ cells and MTAP^+/+^ cells in the presence of an MTAP inhibitor confirm this finding and show a global decrease in SDMA staining on all relevant proteins as established by Western analysis. This indicates that the effect of inhibiting PRMT5 is broad and not specific to any particular protein with the SDMA modification. In the same work, the cytotoxic effect of MTAP inhibition both with and without MAT2A inhibitors was due to inhibition of PRMT5-mediated mRNA splicing resulting in downstream DNA damage^40^.

**Figure 6:**
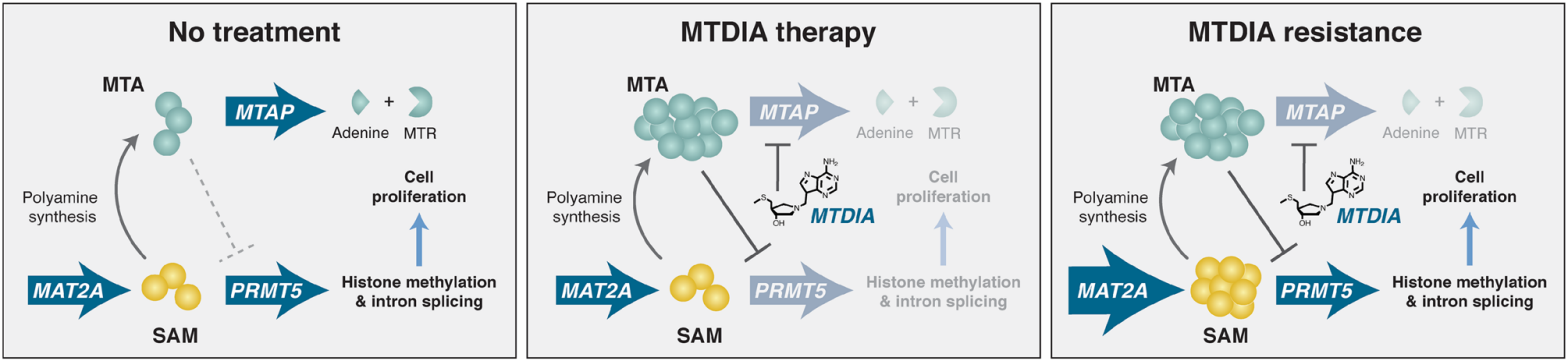
Proposed mechanism of action of MTDIA. In the absence of MTDIA, MTAP metabolizes MTA produced from polyamine synthesis. With MTAP inhibited by MTDIA, MTA accumulates, resulting in competitive inhibition of PRMT5-mediated histone methylation and intron splicing, slowing cancer cell proliferation. In MTDIA resistant cell lines, gene amplification of *MAT2A* increases SAM production to relieve the inhibition caused by excess MTA.

The genetic characteristics of the MTDIA-resistant FaDu cell line that allow it to overcome the effects of inhibited MTAP activity rely on an increased catalytic capacity of MAT2A, via increased *MAT2A* expression. This genetic change provides adequate SAM production to maintain normal cellular methylation functions without cellular depletion of SAM levels, even when SAM salvage from MTA is blocked by MTDIA (Fig. 6), and to overcome the inhibitory effect of accumulated MTA on PRMT5 activity. Resistance to MTAP inhibition conferred by *MAT2A* gene amplification provides increased SAM to overcome MTA inhibition without an increase in MTA production. MTA is produced solely from the polyamine pathway. Treatment with MTDIA has been shown to have no effect on polyamine levels^11,12^.

Previous reports established that deficient methylation potential through methionine restriction leads to decreased tumorigenesis and cancer cell death^41^. Methionine starvation effects can be reversed when cancer cells are supplemented with SAM^42^. The SAM-rescue effect is recapitulated in FaDu-R cells treated with MTDIA. MTDIA resistance occurs as a consequence of increased MAT2A expression to restore the MTAP-inhibited cells with adequate SAM to rescue methylation functions (Fig. 5). The stable metabolic SAM levels in APC^Min/+^ mouse liver following MTDIA treatment, coupled with the MAT2A overexpression providing MTDIA resistance in FaDu-R cells, supports a clear hypothesis for the MTDIA mechanism of action (Fig. 6). This MTA/SAM antagonistic regulation in treated mice is more than simple reversal of competitive inhibition, as the levels of SAM are not altered in the livers of mice treated with MTDIA.

### MTDIA induces MTAP^−/−^ phenotype in MTAP^+/+^ cells

An important anti-cancer application for MTDIA, beyond that implied by the APC^Min/+^ model, is the pharmacological induction of the MTAP^−/−^ physiological state in cancers that are genetically MTAP^+/+^. Approximately 15% of human cancers are genetically deleted in the *MTAP* locus, with deletions in specific cancers (eg. glioblastoma) as frequent as 50%^43^. Synthetic lethal analyses have reported that MTAP^−/−^ cancers are unusually susceptible to agents targeting MAT2A, RIOK1 or PRMT5, thus MAT2A and PRMT5 have been implicated as anti-cancer targets in MTAP^−/−^ cancer cell lines^18–20^. Elevated MTA concentrations in MTAP^−/−^ cancer cells provide partial inhibition of PRMT5, which would be enhanced in terms of cancer cell lethality when combined with an inhibitor of PRMT5 directly or with an inhibitor of MAT2A, which supplies SAM for PRMT5. Current efforts toward MAT2A inhibitor development are focused towards treating MTAP^−/−^ cancers, with agent AG-270 in clinical trials enrolling patients with MTAP-deleted solid tumors (NCT03435250). Results with MTDIA indicate it is sufficient to increase MTA levels comparable to those in cells with genetic inactivation of MTAP. MTAP^−/−^ cell lines, when compared to isogenic MTAP^+/+^ cell lines, have reported MTA levels increased by 1.5- to 5-fold, similar to the 4.2-fold increase in MTA levels seen here (Fig. 3)^19^. Mice dosed with 20 or 30 mg/kg/day MTDIA gave the same MTA levels. Thus, MTAP activity in mice is apparently subject to complete inhibition at either dose of MTDIA, creating a physiologic mimic of MTAP deletion.

The MAT2A inhibitor AG-270 applied to MTAP^−/−^ cancer cells inhibits SAM production, together with increased MTA resulting in decreased SDMA levels as its mechanism of action^44^. The neoplastic cells present in APC^Min/+^ mice are MTAP^+/+^ but are made physiologically MTAP^−/−^ by MTDIA, causing MTA accumulation. The downstream inhibition of PRMT5 is similar to the effects of a MAT2A inhibitor in an MTAP^−/−^ cell line, in that both inhibit cancer cell growth via restriction of PRMT5 activity. SAM sequestration is the mechanism in the case of AG-270 inhibition of MAT2A, and MTA-mediated inhibition of PRMT5 inhibition is the mechanism in the case of MTDIA.

The low toxicity associated with MTDIA therapy at doses well above optimal treatment levels, establishes that a therapeutic window exists to elevate MTA levels in rapidly proliferating cancer cells. The source of MTA production is polyamine synthesis, a pathway known to be upregulated in cancer cells^45^. MTDIA therapy could therefore specifically enhance the sensitivity of cancer cells to PRMT5 or MAT2A inhibitors. The significant investigation into the design of PRMT5 and MAT2A inhibitors in recent years makes the possibility of co-inhibition of MTAP a new possibility for MTAP^+/+^cancers as well as its use as a monotherapy^46,47^.

## Supplementary Information

Supplementary Information
