# Supplementary material for: Transition state analogue of MTAP extends lifespan of APCMin/+ mice
Source: Sci Rep. 2021 Apr 23;11:8844. doi: 10.1038/s41598-021-87734-6 (PMC8065027; doi:10.1038/s41598-021-87734-6)
Supplement: Supplementary file 1 — Supplementary Information [file 41598_2021_87734_MOESM1_ESM.docx]

**Supporting Information**

**Transition State Analogue of MTAP Extends Lifespan of APC^Min/+^ Mice**

Ross S. Firestone^1,2,3^ Mu Feng^1,2^ Indranil Basu,^4^ Karina Peregrina,^5^ Leonard H. Augenlicht^5^ & Vern L. Schramm^1^*

^1^Department of Biochemistry, Albert Einstein College of Medicine, Bronx, NY, USA

^2^Contributed equally to this work.

^3^Current address: Department of Medicine, Icahn School of Medicine at Mount Sinai, New York, NY, USA

^4^Department of Radiation Oncology, Albert Einstein College of Medicine, Bronx, NY, USA

^5^Department of Cell Biology, Albert Einstein College of Medicine, Bronx, NY, USA

*Address correspondence to vern.schramm@einstein.yu.edu

**Contents**

**A. Supporting Information Tables S1-S12**

**B. Supporting Information Figures S1-S5**

**C. Supporting Information Methods**

C1 *Protocol for Tissue Paraffin Embedding and Hematoxylin and Eosin Staining*

C2 *Protocol for Metabolite Measurement by Human Metabolome Technologies*

C3 *Protocol for Preparation of MTDIA Resistant FaDu Cancer Cells*

C4 *Protocol for IHC staining of tumor tissue with anti-SDMA antibodies*

**D. Expanded Metabolomics Data**

**E. Supporting Information References**

**A. Supporting Information Tables S1-S12**

**Table S1.** Median survival of APC^Min/+^ mice and the effect of MTDIA. APC^Min/+^ mouse groups receiving 20 and 30 mg/kg/day oral MTDIA had statistically significant improvements in survival. The most profound effects with the 20 mg/kg/day dose.

|  | **Median Survival (days)** | **P-Value**  **(vs Control)** |
| --- | --- | --- |
| **Control** | 169 | N/A |
| **10 mg/kg/day MTDIA** | 176.5 | 0.72 |
| **20 mg/kg/day MTDIA** | 294 | 0.001 |
| **30 mg/kg/day MTDIA** | 226 | 0.04 |

**Table S2.** MTDIA resistant FaDu cells show four-fold increased copy number of the indicated genes on chromosome 2 when compared to MTDIA sensitive FaDu cells. MAT2A has been implicated as a synthetic lethal anti-cancer target in MTAP-deleted cancer cell lines.

| **Upregulated Gene** | **Gene Function** |
| --- | --- |
| *SUCLG1*^1^ | Succinyl-CoA ligase alpha subunit |
| *DNAH6*^2^ | Dynein axonemal heavy chain |
| *TMSB10*^3^ | Prevents actin polymerization |
| *KCMF1*^4^ | Potassium channel modulation |
| *TCF7L1*^5^ | Wnt signaling |
| *TGOLN2*^6^ | Golgi membrane protein |
| *RETSAT*^7^ | Metabolizes all-trans retinoid acid |
| *ELMOD3*^8^ | Actin / cytoskeleton integrity |
| *CAPG*^9^ | Actin / cytoskeleton integrity |
| *MAT2A* | S-adenosylmethionine synthesis |
| *GGCX^1^*^0^ | Vitamin K dependent protein activation |
| *VAMP5/VAMP8*^11^ | Vesicle docking and fusion |
| *RNF181*^12^ | Ubiquitin E3 ligase |
| *USP39*^13^ | Splices aurora-B mRNA to increase expression |

**Table S3.** Toxicity test doses of MTDIA and MTA for each test group (8 groups of n = 5). Groups A-D tested the toxicity of high doses of MTDIA alone. Groups E-H tested the potential toxic effects of supplemental MTA on mice receiving high MTDIA doses. Daily i.p. dose values were calculated to a mg/kg/day basis for individual mouse cohorts based on body weights.

| **Group** | **Daily i.p. MTDIA dose (mg/kg/day)** | **Daily i.p. MTA dose**  **(mg/kg/day)** |
| --- | --- | --- |
| **A** | 0 | 0 |
| **B** | 57.1 | 0 |
| **C** | 169.6 | 0 |
| **D** | 316.9 | 0 |
| **E** | 0 | 0 |
| **F** | 170 | 17 |
| **G** | 169 | 34 |
| **H** | 176 | 65 |

**Table S4.** Weekly body weights of each group of mice from the toxicity experiment (Table S3). No group experienced weight loss of greater than 8% initial body weight for MTDIA doses of up to 15 times the optimal therapeutic dose of 20 mg/kg/day. MTA supplementation of MTDIA-treated mice also showed no apparent toxic effects. Asterisk (*) symbol indicates that one mouse in the indicated group died of undiagnosed causes during the indicated week.

| **Group** | **Number of Mice** | **Average Weight Day 0** | **Average Weight Week 1** | **Average Weight Week 2** | **Average Weight Week 3** | **Average Weight Week 4** |
| --- | --- | --- | --- | --- | --- | --- |
| **A** | 4 | 29.7 ± 1.1 | 29.3 ± 1.9 | 27.7 ± 1.1 | 28.1 ± 1.4 | 28.5 ± 1.6 |
| **B** | 5 | 31.1 ± 1.4 | 30.2 ± 1.8 | 29.5 ± 1.4 | 30.2 ± 1.7 | 30.5 ± 1.5 |
| **C** | 5 | 31.5 ± 2.5 | 30.8 ± 2.3 | 29.9 ± 2.2 | 30.1 ± 1.9 | 30.8 ± 1.7 |
| **D** | 3 | 33.0 ± 0.9 | 30.2 ± 1.5 | 29.9 ± 1.1 | 30.1 ± 1.1 | 30.3 ± 1.5 |
| **E** | 4 | 32.2 ± 4.1 | 30.4 ± 3.3 | 30.9 ± 2.9 | 30.5 ± 2.4* | 29.9 ± 2.5 |
| **F** | 5 | 33.1 ± 2.5 | 30.7 ± 2.4 | 30.2 ± 2.8 | 29.3 ± 2.1 | 29.3 ± 1.7 |
| **G** | 5 | 32.2 ± 1.6 | 29.8 ± 1.6 | 30.5 ± 1.0 | 30.3 ± 0.8 | 31.0 ± 1.1 |
| **H** | 5 | 30.0 ± 1.4 | 28.5 ± 1.6 | 29.3 ± 1.0 | 29.6 ± 1.1 | 30.0 ± 1.7* |

**Table S5.** Tumor size and number in APC^Min/+^ untreated (Control) or Treated (20 mg/kg) mice at 150 days of age (as in Table S1 and Figure 2). Tumor size and tumor number was determined from microscopic examination of histologic sections from Swiss rolls fixed with H&E as described in the methods. The data are from three individual mice from each group. Tumor size is in equivalent but arbitrary units.

| **Mean Tumor Size (AU)** | | **Number of Tumors** | |
| --- | --- | --- | --- |
| **Control** | **Treated** | **Control** | **Treated** |
| 239 | 236 | 8 | 9 |
| 284 | 134 | 6 | 7 |
| 323 | 297 | 6 | 7 |
| 279 | 176 | 4 | 6 |
| 332 | 109 | 3 | 3 |
| 453 | 128 | 2 | 2 |
| **Avg 318 ± 74** | **Avg 180 ± 73** | **Avg 4.8 ± 2.2** | **Avg 5.7 ± 2.7** |

**B. Supporting Information Figures S1-S4**

**Figure S1:** The design of (A) Study A and (B) Study B. Study A determined if oral MTDIA improved survival in APC^Min/+^ mice. Three treatment doses were compared to control mice with animals being given oral MTDIA dissolved in their drinking water from weaning at 21 days. Study B compared tumor burden via histological analysis (N=6 in each group) liver metabolomics (N = 6 in each group) and routine blood work analysis (N=8 control and N=6 treated mice) in 150 day-old mice.

**Figure S2:** APC^Min/+^ mouse survival study. Time to death for untreated APC^Min/+^ mice is consistent with previous reports. Mice treated with 10 mg/kg/day showed no improvement in survival. Mice treated with 20 mg/kg/day receiving the optimal dose for therapy with median survival increasing nearly two-fold (See Table S1). Mice treated with 30 mg/kg/day MTDIA showed improved survival but less than for the 20 mg/kg/day group.

**Figure S3:** Quantitative data from individual mice from IHC staining with anti-SDMA antibodies. The values shown represent the percent of the encircled area from the selected region of interest that stained positive for SDMA in each individual mouse. The four control mice shown have universally higher levels of observed SDMA compared to all individual treated mice.

**Figure S4:** The relative genomic levels of genes across all chromosomes in MTDIA-resistant FaDu-R cells relative to FaDu WT cells (A). Chromosome 2 shows one region with a significant gene amplification. Its expanded form (B) demonstrates a four-fold gene amplification containing several encoding regions (See Table SA2). For the MTDIA target, the most relevant is *MAT2A*. Quantitative PCR and Western blotting experiments confirmed overexpression of mRNA and MAT2A in FaDu-R cells.

**Figure S5:** Unedited western blot for data represented in Figure 5. The first panel represents shows the relative amounts of MAT2A in FaDu Wild-Type (WT) vs MTDIA resistant FaDu cells (R). The second panel shows the relative amounts of MAT2B, and the third panel shows the presence of Beta-actin. Unlabeled lanes were related to separate experiments not discussed in this manuscript.

**C. Supporting Information Methods**

**C1. *Protocol for Tissue Histopathology Preparation and Hematoxylin and Eosin Staining***

Following tissue fixation and paraffin embedding, tissue was sectioned and mounted in preparation for staining. Slides were deparaffinized and stained with hematoxylin and eosin according to the protocol in table S6.

**Table S6.** For hematoxylin and eosin staining slides were immersed in sequential solutions as indicated. “Acid Alcohol 1%” solution contains 6 mL of 12.5 N HCl mixed with 600 mL of 70% Alcohol. “Ammonia Solution” contains 1.5 mL of 28% ammonium hydroxide in 600 mL H_2_O.

| **Step #** | **Solution** | **Time** |
| --- | --- | --- |
| 1 | Xylene | 8 minutes |
| 2 | Xylene | 7 minutes |
| 3 | 100% Alcohol | 3 minutes |
| 4 | 100% Alcohol | 3 minutes |
| 5 | 95% Alcohol | 2 minutes |
| 6 | 80% Alcohol | 2 minutes |
| 7 | 70% Alcohol | 1 minute |
| 8 | Wash | 2 minutes |
| 9 | Hematoxylin | 8 minutes |
| 10 | Wash | 10 seconds |
| 11 | Wash | 5 minutes |
| 12 | Acid Alcohol 1% | 5 seconds |
| 13 | Wash | 3 minutes |
| 14 | Ammonia Solution | 10 seconds |
| 15 | Wash | 4 minutes |
| 16 | 80% Alcohol | 1 minute |
| 17 | Eosin | 8 minutes |
| 18 | 100% Alcohol | 1 minute |
| 19 | 100% Alcohol | 1 minute |
| 20 | 100% Alcohol | 1 minute |
| 21 | Xylene | 2 minutes |
| 22 | Xylene | 5 minutes |
| 23 | Xylene | 5 minutes |

**C2. *Protocol for Metabolite Measurement by Human Metabolome Technologies***

C2.1 Materials

Targeted quantitative analysis was performed on 15 samples of mouse liver (Table S7) using capillary electrophoresis mass spectrometry (CE-TOFMS and CE-QqQMS) in the cation and anion analysis modes. A total of 116 metabolites (54 and 62 metabolites in the cation and anion mode, respectively) involved in glycolysis, pentose phosphate pathway, tricarboxylic acid (TCA) cycle, urea cycle, polyamine, creatine, purine, glutathione, nicotinamide, choline, and amino acid metabolisms were annotated based on the HMT metabolite database. In addition, 5'-methylthioadenosine was analyzed.

**Table S7. Tissue Samples**

| Mouse Number | Liver Mass  (mg) | Treatment Group | Dilution (Cation) | Dilution (Anion) |
| --- | --- | --- | --- | --- |
| 1 | 53.5 | Control | 1 | 3 |
| 2 | 38.8 |  | 1 | 3 |
| 3 | 37.8 |  | 1 | 3 |
| 4 | 47.3 |  | 1 | 3 |
| 5 | 43.0 |  | 1 | 3 |
| 6 | 46.8 |  | 1 | 3 |
| 7 | 48.0 | 20 mg/kg/day | 1 | 3 |
| 8 | 45.9 |  | 1 | 3 |
| 9 | 43.8 |  | 1 | 3 |
| 10 | 39.1 |  | 1 | 3 |
| 11 | 39.4 |  | 1 | 3 |
| 12 | 39.5 |  | 1 | 3 |
| 13 | 45.7 | 30 mg/kg/day | 1 | 3 |
| 14 | 42.8 |  | 1 | 3 |
| 15 | 37.6 |  | 1 | 3 |

C2.2 Tissue Preparation

Tissues were sent from the Albert Einstein College of Medicine (AECOM) to Human Metabolome Technologies (HMT). At HMT, the samples were mixed with 1,500 μL of 50% acetonitrile in water (v/v) containing internal standards (10 μM) and homogenized by a homogenizer (1,500 rpm, 120 sec x 3 times). The supernatant (400 μL) was then filtrated through 5-kDa cut-off filter (ULTRAFREE-MC-PLHCC, Human Metabolome Technologies, Yamagata, Japan) to remove macromolecules. The filtrate was centrifugally concentrated and resuspended in 50 μL of ultrapure water immediately before analysis.

C2.3 Cation Analysis

Cationic compounds were analyzed as shown in Table S8 based on established methods.^14-16^ The samples were diluted as shown in Table S7 for CE-TOFMS analysis.

**Table S8: Conditions for Cation Analysis**

| Device | |
| --- | --- |
| CE-TOFMS | Agilent CE-TOFMS system (Agilent Technologies) Machine No. 8 |
| Capillary | Fused silica capillary, i.d. 50 μM × 80 cm |
| Analytical Condition | |
| Run Buffer | Cation buffer solution (p/n: H3301-1001) |
| Rinse Buffer | Cation buffer solution (p/n: H3301-1001) |
| Sample Injection | Pressure injection at 50 mbar, 5s |
| CE Voltage | Positive 30 kV |
| MS Ionization | ESI Positive |
| MS Capillary Voltage | 4,000 V |
| MS Scan Range | *m/z* 50-1,000 |
| Sheath Liquid | HMT sheath liquid (p/n: H3301-1020) |

C2.4 Anion Analysis

Anionic compounds were analyzed using CE-MS/MS as shown in Table S9 based on established methods.^14-16^ The samples were diluted as shown in Table S7 for CE-QqQMS analysis.

**Table S9. Conditions for Anion Analysis**

| Device | |
| --- | --- |
| CE | Agilent CE system |
| MS | Agilent 6460 TripleQuad LC/MS Machine No. QqQ1 |
| Capillary | Fused silica capillary, i.d. 50 μM × 80 cm |
| Analytical Condition | |
| Run Buffer | Anion buffer solution (p/n: H3302-1021) |
| Rinse Buffer | Anion buffer solution (p/n: H3302-1021) |
| Sample Injection | Pressure injection at 50 mbar, 5s |
| CE Voltage | 30 kV |
| MS Ionization | ESI positive and negative |
| MS Capillary Voltage | 4,000 V for Positive and 3500V for negative modes |
| Sheath Liquid | HMT sheath liquid (p/n: I3300-1030) |

C2.5 Data Processing

Peaks detected in CE-TOFMS analysis were extracted using automatic integration software

(MasterHands ver.2.17.1.11 developed at Keio University)^17^ and those in CE-QqQMS analysis were extracted using automatic integration software (MassHunter Quantitative Analysis B.06.00 Agilent Technologies, Santa Clara, CA, USA) in order to obtain peak information including *m/z*, migration time (MT), and peak area. The peak area was then converted to relative peak area (equation 1). The peaks were annotated based on migration times in CE and *m*/*z* values determined by TOFMS.

Equation 1. $\text{Relative Peak Area}=\frac{\text{Metabolite Peak } \text{Area}}{\text{Internal Standard Peak Area }\times\text{ Normalization Factor}^{\text{*}}}$

*Normalization factor is calculated if the volume or amount of samples used in the analysis is varied depending on the sample

Putative metabolites were then assigned from HMT metabolite database on the basis of *m/z* and MT. The tolerance was ±0.5 min in MT and ±10 ppm in *m/z* (Equation 2)

Equation 2. $\text{Mass error (ppm) = }\frac{\text{Measured Value - Theoretical Value}}{\text{Measured Value}}\times10^{6}$

In addition, absolute quantification was performed in 116 metabolites including glycolytic and TCA cycle intermediates, amino acids, and nucleic acid precursors. All metabolite concentrations were calculated by normalizing the peak area of each metabolite with respect to the area of the internal standard and by using standard curves, which were obtained by three-point calibrations. 5′-Methylthioadenosine concentrations (methylthioadenosine in Table S12) were calculated by normalizing the peak area of each metabolite with respect to the area of the internal standard and by using standard curves, which were obtained by single-point (100 μM) calibrations.

C2.6. Statistical Analysis

Hierarchical cluster analysis (HCA) and principal component analysis (PCA) were performed by

statistical analysis software (developed at HMT). The analysis results were shown in Table S12.

**C3. *Protocol for Preparation of MTDIA Resistant FaDu Cancer Cells***

Surviving cells cultured under increasing drug pressure were passaged every 3 days. The selection process took 102 days to convert FaDu (WT) cells to FaDu-R cells, resistant to 20 μM MTA + 1 μM MTDIA in culture media (see Table S10)*.* From passage 33 and onward, cells were grown in the presence of 20 μM MTA + 1 μM MTDIA in culture dishes when thawed from frozen stocks. The resistant cells grew at equivalent rates to WT cells when cultured in the presence of 20 μM MTA + 1 μM MTDIA. No changes in morphology (increase in size etc.) were observed in FaDu-R cells compared to FaDu WT cells.

**Table S10:** The FaDu culture media contents and number of passages for resistance are indicated below. A total of 33 passages over 102 days were necessary to generate the MTIDIA-resistant FaDu-R line.

| **Passage Numbers** | **Concentration of MTA + MTDIA** |
| --- | --- |
| 1 – 4 | 5 μM MTA + 0.5 μM MTDIA |
| 5 – 14 | 10 μM MTA + 0.5 μM MTDIA |
| 15 – 19 | 10 μM MTA + 1 μM MTDIA |
| 20 – 24 | 15 μM MTA + 1 μM MTDIA |
| 25 – 33 | 20 μM MTA + 1 μM MTDIA |

**C4. *Protocol for IHC staining of tumor tissue with anti-SDMA antibodies***

Immunohistochemistry (IHC) samples were prepared as previously reported.^18^ Mouse intestinal tissue from 11 mice from Study B (Figure S1) were euthanized and intestinal tissue was formalin-fixed and prepared as intestinal “Swiss-rolls” as described previously. Individual sections were then deparaffinized and stained by incubating at 60 °C for 1 hour, following the stepwise protocol described in Table S11. Following staining, sections were developed using a Vectastain Elite ABC Kit (Vector Lab) and a DAB Quanto kit (Thermo Scientific) followed by a light counterstain with hematoxylin. Slides were then mounted in Permount (Fisher Scientific).

Stained samples were analyzed under a light microscope (DP73, Olympus) as shown in Figure 4A. Individual positively stained regions of interest (ROIs) were selected by an operator blinded to sample group assignments, and the percentage of positively stained areas in each ROI were calculated. Three regions of interest were selected from nine sections of intestine per mouse (proximal, middle and distal regions of the duodenum, jejunum and ileum). Regions of interest were circular and were each ~16 micrometers in diameter, typically encircling 6-8 cells on average. The signal intensity in each ROI was averaged for within each individual mouse (Figure 4B). Statistical analysis was performed by comparing signal intensity in treatment and control groups using a two-tailed Student’s *t* test.

**Table S11:** IHC staining protocol. Anti-SDMA Ab was purchased from MyBioSource (Cat. #MBS619480). Biotinylated goat anti-rabbit IgG was purchased from Vector Lab (Cat. # BA-1000)

| **Step #** | **Solution** | **Time** |
| --- | --- | --- |
| 1 | Xylene | 5 minutes |
| 2 | Xylene | 5 minutes |
| 3 | 100% Ethanol | 8 minutes |
| 4 | 95% Ethanol | 4 minutes |
| 5 | 80% Ethanol | 4 minutes |
| 6 | 70% Ethanol | 5 minutes |
| 7 | 50% Ethanol | 5 minutes |
| 8 | Water | 10 seconds |
| 9 | Water | 5 minutes |
| 10 | 3% H_2_O_2_ in 50% Methanol | 30 minutes |
| 11 | 10 mM sodium citrate (pH = 6.0) | 10 minutes (sub-boiling) |
| 12 | Water | 10 minutes |
| 13 | 5% Goat Serum PBST | 1 hour |
| 14 | 1:400 anti-SDMA Ab | 1 hour (room temp) |
| 15 | 1:400 anti-SDMA Ab | Overnight (4 °C) |
| 16 | 1:100 biotinylated goat anti-rabbit IgG | 30 minutes |

**D. Expanded Metabolomics Data**

**Table S12.** The absolute concentrations of 117 metabolites from mouse liver tissue samples are shown. Values labeled “N/A” indicate that data was insufficient to quantitate this metabolite. A p value of NS indicates values >0.1, a single asterisk (*) indicates P < 0.05, and two asterisks (**) indicate P < 0.01. Statistical significance was calculated using Welch’s t-test.

| **Metabolite** | **Control**  **(A) (nmol/g)** | **20 mg/kg/day**  **(B) (nmol/g)** | **30 mg/kg/day**  **(C) (nmol/g)** | **P value**  **(A vs B)** | **P value**  **(B vs C)** |
| --- | --- | --- | --- | --- | --- |
| NAD+ | 161 ± 30 | 198 ± 60 | 288 ± 47 | NS | NS |
| cAMP | 0.6 ± 0.2 | 0.7 ± 0.3 | 0.7 ± 0.1 | NS | N/A |
| cGMP | 0.013 ± 0.002 | 0.013 ± 0.002 | N/A | NS | NS |
| NADH | 3.6 ± 0.6 | 3.7 ± 0.5 | 4.0 ± 0.5 | NS | NS |
| Xanthine | 99 ± 64 | 87 ± 42 | 103 ± 14 | NS | NS |
| ADP-ribose | 130 ± 56 | 134 ± 24 | 153 ± 26 | NS | NS |
| Mevalonic acid | N/A | N/A | N/A | N/A | NS |
| UDP-glucose | 167 ± 95 | 234 ± 131 | 156 ± 123 | NS | NS |
| Uric Acid | 4.3 ± 1.8 | 4.7 ± 2.3 | 5.2 ± 2.4 | NS | NS |
| NADP+ | 66 ± 34 | 80 ± 45 | 98 ± 19 | NS | NS |
| IMP | 75 ± 44 | 115 ± 73 | 169 ± 47 | NS | NS |
| Sedoheptulose 7-phosphate | 170 ± 77 | 151 ± 61 | 110 ± 17 | NS | NS |
| Glucose-6-phosphate | 419 ± 47 | 418 ± 100 | 327 ± 73 | NS | 0.021* |
| Fructose-6-phosphate | 127 ± 15 | 101 ± 19 | 64 ± 14 | 0.025* | NS |
| Fructose-1-phosphate | 60 ± 15 | 56 ± 17 | 56 ± 14 | NS | NS |
| Galactose 1-phosphate | 34 ± 24 | 28 ± 17 | 32 ± 15 | NS | NS |
| Glucose 1-phosphate | 35 ± 6.6 | 34 ± 14 | 24 ± 8 | NS | N/A |
| Acetoacetyl CoA | N/A | N/A | N/A | N/A | NS |
| Acetyl CoA | 3.9 ± 2.4 | 4.8 ± 3.4 | 9.4 ± 2.7 | NS | NS |
| Folic acid | 0.15 ± 0.01 | 0.2 ± 0.02 | 0.2 ± 0.03 | NS | NS |
| Ribose 5-phosphate | 78 ± 23 | 95 ± 30 | 74 ± 17 | NS | NS |
| CoA | 108 ± 25 | 118 ± 35 | 135 ± 13 | NS | NS |
| Ribose 1-phosphate | 162 ± 24 | 164 ± 35 | 181 ± 13 | NS | NS |
| Ribulose 5-phosphate | 55 ± 13 | 42 ± 20 | 21 ± 7 | NS | NS |
| Xylulose 5-phosphate | 30 ± 4 | 28 ± 14 | 18 ± 8 | NS | NS |
| Erythrose 4-phosphate | 0.4 ± 0.3 | 1.0 ± 0.3 | 0.5 ± 0.3 | 0.013* | NS |
| HMG CoA | 0.2 ± 0.1 | 0.2 ± 0.1 | 0.3 ± 0.1 | NS | NS |
| Glyceraldhyde 3-phosphate | 4.2 ± 2.8 | 2.8 ± 1.0 | 1.5 ± 0.8 | NS | NS |
| NADPH | 3.0 ± 0.6 | 3.2 ± 0.8 | 3.1 ± 0.3 | NS | N/A |
| Malonyl CoA | 0.2 ± 0.1 | 0.2 ± 0.1 | 0.2 ± 0.1 | NS | NS |
| Phosphocreatine | 1.0 ± 0.6 | 1.0 ± 0.5 | 1.7 ± 0.6 | NS | NS |
| XMP | 0.2 ± 0.1 | 0.2 ± 0.01 | 0.2 ± 0.05 | NS | NS |
| Dihydroxyacetone phosphate | 104 ± 66 | 76 ± 23 | 55 ± 30 | NS | NS |
| Adenylosuccinic acid | 68 ± 31 | 58 ± 33 | 24 ± 8 | NS | NS |
| Fructose 1,6-diphosphate | 11 ± 4 | 11 ± 3.6 | 7.5 ± 0.8 | NS | 0.025* |
| 6-Phosphogluconic acid | 117 ± 22 | 116 ± 18 | 93 ± 6 | NS | NS |
| *N*-Carbamoylaspartic acid | 1.8 ± 1.9 | 1.8 ± 1.9 | 0.5 ± 0.3 | NS | NS |
| PRPP | 3.4 ± 0.8 | 4.0 ± 1.2 | 4.6 ± 1.9 | NS | 0.04* |
| 2-Phophoglyceric acid | 0.8 ± 0.1 | 0.9 ± 0.1 | 0.7 ± 0.1 | NS | NS |
| 2,3-Diphosphoglyceric acid | 37 ± 12 | 53 ± 26 | 61 ± 7 | NS | NS |
| 3-Phosphoglyceric acid | 7.3 ± 1.4 | 8.1 ± 1.2 | 5.3 ± 1.5 | NS | NS |
| Phosphonolpyruvic acid | 1.2 ± 0.4 | 1.4 ± 0.3 | 0.7 ± 0.5 | NS | NS |
| GMP | 200 ± 73 | 250 ± 70 | 260 ± 18 | NS | NS |
| AMP | 898 ± 437 | 1,201 ± 499 | 1261 ± 122 | NS | N/A |
| 2-Oxolsovaleric acid | N/A | N/A | N/A | N/A | NS |
| GDP | 29 ± 2.4 | 33 ± 4.5 | 30 ± 5 | NS | NS |
| Lactic Acid | 13,457 ± 2,936 | 11,959 ± 1,842 | 14,306 ± 1,396 | NS | NS |
| ADP | 465 ± 320 | 590 ± 140 | 527 ± 73 | 0.085 | NS |
| GTP | 31 ± 18 | 26 ± 9.6 | 22 ± 10 | NS | N/A |
| Glyoxylate | N/A | N/A | N/A | N/A | NS |
| ATP | 492 ± 320 | 450 ± 224 | 331 ± 131 | NS | NS |
| Glycerol 3-phosphate | 1,450 ± 307 | 1,437 ± 172 | 1,424 ± 236 | NS | NS |
| Glycolic acid | 8.5 ± 3.4 | 9.6 ± 3.8 | 19 ± 10 | NS | NS |
| Pyruvic acid | 122 ± 43 | 159 ± 115 | 148 ± 101 | NS | NS |
| *N*-Acetylglutamic acid | 92 ± 63 | 73 ± 41 | 48 ± 32 | NS | NS |
| 2-Hydroxyglutaric acid | 18 ± 5 | 20 ± 7 | 27 ± 4 | NS | N/A |
| Carbamoylphosphate | N/A | N/A | N/A | N/A | NS |
| Succinic acid | 1,477 ± 237 | 1,272 ± 258 | 987 ± 115 | NS | NS |
| Malic acid | 714 ± 276 | 599 ± 511 | 160 ± 130 | NS | N/A |
| 2-Oxoglutaric acid | N/A | N/A | N/A | N/A | NS |
| Fumaric acid | 243 ± 72 | 198 ± 129 | 83 ± 26 | NS | 0.008** |
| Citric acid | 100 ± 32 | 92 ± 23 | 54 ± 5 | NS | 0.047* |
| *cis*-Acotinic acid | 1.0 ± 0.4 | 0.9 ± 0.4 | 0.4 ± 0.1 | NS | N/A |
| Isocitric acid | N/A | N/A | N/A | N/A | 0.023* |
| Urea | 9,694 ± 3,355 | 9,990 ± 2,233 | 6,973 ± 854 | NS | NS |
| Glycine | 1512 ± 231 | 1,590 ± 498 | 1,819 ± 289 | NS | NS |
| Putrescine | 8.0 ± 3.0 | 6.0 ± 2.3 | 4.4 ± 1.2 | NS | NS |
| Sarcosine | 112 ± 88 | 108 ± 54 | 90 ± 10 | NS | NS |
| β-Alanine | 386 ± 118 | 275 ± 141 | 180 ± 27 | NS | NS |
| Alanine | 4,809 ± 1299 | 4,056 ± 1,048 | 3,549 ± 45 | NS | NS |
| GABA | 44 ± 14 | 41 ± 29 | 36 ± 6 | NS | 0.056 |
| *N,N*-Dimethylglycine | 57 ± 30 | 40 ± 11 | 23 ± 9 | NS | NS |
| Choline | 33 ± 7 | 51 ± 21 | 51 ± 30 | NS | 0.085 |
| Serine | 282 ± 75 | 259 ± 59 | 320 ± 31 | NS | NS |
| Camosine | 0.9 ± 0.2 | 0.8 ± 0.2 | 1.0 ± 0.3 | NS | 0.092 |
| Creatinine | 5.4 ±0.7 | 5.0 ± 1.2 | 3.5 ± 0.9 | NS | NS |
| Proline | 565 ± 450 | 495 ± 316 | 368 ± 72 | NS | NS |
| Betaine | 700 ± 447 | 420 ± 208 | 358 ± 52 | NS | NS |
| Valine | 373 ± 84 | 384 ± 117 | 445 ± 55 | NS | NS |
| Homoserine | 6.7 ± 2.3 | 4.9 ± 2.0 | 4.1 ± 1.1 | NS | 0.085 |
| Threonine | 460 ± 78 | 412 ± 70 | 502 ± 54 | NS | NS |
| Betaine aldehyde | 0.6 ± 0.5 | 0.6 ± 0.4 | 1.1 ± 0.6 | NS | 0.083 |
| Cysteine | 28 ± 16 | 30 ± 13 | 12 ± 11 | NS | NS |
| Hyroxyproline | 33 ± 16 | 38 ± 34 | 25 ± 22 | NS | NS |
| Creatine | 321 ± 52 | 298 ± 58 | 278 ± 46 | NS | NS |
| Isoleucine | 196 ± 44 | 195 ± 70 | 221 ± 38 | NS | NS |
| Leucine | 367 ± 79 | 382 ± 126 | 430 ± 54 | NS | NS |
| Asparagine | 167 ± 21 | 161 ± 34 | 147 ± 14 | NS | NS |
| Ornithine | 314 ± 58 | 298 ± 137 | 256 ± 51 | NS | NS |
| Aspartic acid | 825 ± 369 | 773 ± 493 | 426 ± 34 | NS | N/A |
| Homocysteine | N/A | N/A | N/A | N/A | NS |
| Adenine | 3.7 ± 0.8 | 3.5 ± 0.9 | 2.6 ± 1.5 | NS | NS |
| Hypoxanthine | 166 ± 41 | 152 ± 73 | 128 ± 21 | NS | NS |
| Spermidine | 18 ± 3 | 21 ± 6 | 32 ± 13 | NS | NS |
| Glutamine | 3,008 ± 480 | 2,935 ± 614 | 3,044 ± 393 | NS | NS |
| Lysine | 871 ± 229 | 753 ± 218 | 646 ± 82 | NS | 0.052 |
| Glutamatic acid | 2,903 ± 942 | 2,846 ± 1,265 | 1,536 ± 207 | NS | NS |
| Methionine | 94 ± 52 | 78 ± 25 | 59 ± 5 | NS | N/A |
| Guanine | 1.6 ± 0.9 | 2.5 ± 1.3 | N/A | NS | NS |
| Histidine | 655 ± 95 | 604 ± 60 | 589 ± 16 | NS | 0.036* |
| Carnitine | 269 ± 74 | 243 ± 98 | 129 ± 9 | NS | NS |
| Phenylalanine | 167 ± 37 | 166 ± 42 | 145 ± 26 | NS | NS |
| Arginine | 1.7 ± 0.5 | 1.6 ± 0.3 | 1.9 ± 0.3 | NS | NS |
| Citrulline | 81 ± 35 | 59 ± 21 | 58 ± 12 | NS | NS |
| Tyrosine | 221 ± 41 | 266 ± 63 | 208 ± 6 | NS | NS |
| *S*-Adenosylhomocysteine | 54 ± 21 | 68 ± 20 | 52 ± 2 | NS | NS |
| Spermine | 11 ± 2 | 13 ± 4.5 | 18 ± 11 | NS | NS |
| Tryprophan | 61 ± 13 | 64 ± 15 | 65 ± 4 | NS | NS |
| Cystathionine | 120 ± 85 | 81 ± 57 | 81 ± 31 | NS | NS |
| Adenosine | 583 ± 308 | 494 ± 349 | 223 ± 23 | NS | NS |
| Inosine | 831 ± 217 | 671 ± 308 | 458 ± 46 | NS | NS |
| Guanosine | 142 ± 80 | 102 ± 66 | 41 ± 7 | NS | NS |
| Argininosuccinic acid | 7.0 ± 1.3 | 6.0 ± 1.9 | 5.5 ± 1.3 | NS | 0.032* |
| Glutathione (GSSG) | 416 ± 84 | 544 ± 117 | 393 ± 53 | 0.056 | NS |
| Glutathione (GSH) | 6,373 ± 1910 | 7,176 ± 1,620 | 7,224 ± 593 | NS | 0.047* |
| *S-*Adenosylmethionine | 90 ± 42 | 84 ± 15 | 68 ± 4 | NS | NS |
| Methylthioadenosine (MTA) | 1.0 ± 0.6 | 4.2 ± 1.3 | 4.2 ± 0.7 | 0.001** | 0.057 |

**E. Supporting Information References**

1. Ostergaard, E.; Chritensen, E.; Kristensen, E.; Mogensen, B.; Duno, M.; Shoubridge, E.A.; Wibrand, F. *Am J Hum Genet*. **2007**, *81*, 383-387.

2. Li, Y.; Yagi, H.; Onuoha, E.O.; Damerla, R.R.; Francis, R.; Furutani, Y.; Tariq, M.; King, S.M.; Hendricks, G.; Cui, C.; Saydmohammed, M.; Lee, D.M.; Zahid, M.; Sami, I.; Leatherbury, L.; Pazour, G.J.; Ware, S.M.; Nakanishi, T.; Goldmuntz, E.; Tsang, M.; Lo, C.W. *PLoS Genet.* **2016**, *12*, e1005821

3. Song, C.; Su, Z.; Guo, J. *Biosci Rep*. **2019**, *39.*

4. Beilke, S.; Oswald, F.; Genze, F.; Wirth, T.; Adler, G.; Wagner, M. *Oncogene*. **2010**, *29*, 4058-4067.

5. Korinek, V.; Barkerm N.; Willert, K.; Molenaar, M.; Roose, J.; Wagenaar, G.; Markman, M.; Lamers, W.; Destree, O.; Clevers, H. Mol Cell Biol. **1998**, 18, 1248-1256.

6. Ponnambalam, S.; Girotti, M.; Yaspo, M.L.; Owen, C.E.; Perry, A.C.; Suganuma, T.; Nilsson, T.; Fried, M.; Banting, G.; Warren G. *J Cell Sci*. **1996**, *109*, 675-685.

7. Pang, X.Y.; Wang, S.; Jurczak, M.J.; Shulman, G.I.; Moise, A.R. *Arch Biochem Biophys*. **2017**, *633*, 93-102.

8. Li, W.; Sun, J.; Ling, J.; Li, J.; He, C.; Liu, Y.; Chen, H.; Men, M.; Niu, Z.; Deng, Y.; Li, M.; Li, T.; Wen, J.; Sang, S.; Li, H.; Wan, Z.; Richard, E.M.; Chapagain, P.; Yan, D.; Liu, X.Z.; Mei, L.; Feng, Y. *Hum Genet*. **2018**, *137*, 329-342.

9. Watari, A.; Takaki, K.; Higashiyama, S.; Li, Y.; Satomi, Y.; Takao, T.; Tanemura, A.; Yamaguchi, Y.; Katayama, I.; Shimakage, M.; Miyashiro, I.; Takami, K.; Kodama, K.; Yutsudo, M. *Oncogene*. **2006**, *25*, 7373-7380.

10. Lin, P.J.; Jin, D.Y.; Tie, J.K.; Presnell, S.R.; Straight, D.L.; Stafford, D.W. *J Biol Chem*. **2002**, *277*, 28584-28591.

11. Söllner, T.; Bennett, M.K.; Whiteheart, S.W.; Scheller, R.H.; Rothman, J.E. *Cell*. **1993**, *75*, 409-418.

12. Brophy, T.R.; Raab, M.; Daxecker, H.; Culligan, K.G.; Legmann, I.; Chubb, A.J.; Treumann, A.; Moran N. *Biochem Biophys Res Commun*. **2008**, *369*, 1088-1093.

13. van Leuken, R.J.; Luna-Vargas, M.P.; Sixma, T.K.; Wolthuis, R.M.; Medema, R.H. *Cell Cycle*. **2008**, *7*, 2710-2719.

14. Soga, T.; Heiger, D.N. *Anal Chem*. **2000**, *72*, 1236-1241.

15. Soga, T.; Ueno, Y.; Naraoka, H.; Ohashi, Y.; Tomita, M.; Nishioka, T. *Anal Chem*. **2002**, *74*, 2233-2239.

16. Soga, T.; Ohashi, Y.; Ueno, Y.; Naraoka, H.; Tomita, M.; Nishioka, M. *J Proteome Res.* **2003**, *2*, 488-494.

17. Sugimoto, M.; Wong, D.T.; Hirayama, A.; Soga, T.; Tomita, M. *Metabolomics*. **2010**, *6*, 78-95.

18. Wang, D.; Peregrina, K.; Dhima, E.; Lin, E.Y.; Mariadason, J.M.; Augenlicht, L.H. *Proc Natl Acad Sci U S A*. **2011**, *108*, 10272-10277
